# Is race-specific neighborhood social cohesion key to reducing racial disparities in late HIV diagnosis: A multiyear ecological study

**DOI:** 10.1016/j.sste.2022.100508

**Published:** 2022-04-20

**Authors:** Yusuf Ransome, Hui Luan, Lorraine T Dean, Harrison Quick, Tanner Nassau, Ichiro Kawachi, Kathleen A Brady

**Affiliations:** a Department of Social and Behavioral Sciences, Yale School of Public Health, 60 College Street, LEPH 4th Floor, New Haven, CT 06520; b Department of Geography, Spatial Cognition, Computation, and Complexity (S3C) Lab, University of Oregon, 107D Condon Hall, 1251 University of Oregon, Eugene OR, 97403; c School of Geodesy and Geomatics, Wuhan University, 129 Luoyu Road, Wuchang District, Wuhan, Hubei, China, 430079; d Department of Epidemiology, Johns Hopkins Bloomberg School of Public Health, 615N Wolfe St, E6650, Baltimore, MD, 21205; e Department of Epidemiology and Biostats, Drexel University Dornsife School of Public Health, 3215 Market St, Philadelphia, PA, 19104; f AIDS Activities Coordinating Office, Philadelphia Department of Public Health, 1101 Market St., 8th Floor, Philadelphia, PA, 19107; g Department of Social and Behavioral Sciences, Harvard T.H. Chan School of Public Health, 677 Huntington Avenue, Kresge Building 7th Floor, Boston MA, 02115; h AIDS Activities Coordinating Office, Philadelphia Department of Public Health, 1101 Market St., 8th Floor, Philadelphia, PA, 19107

**Keywords:** HIV/AIDS, social capital, social cohesion, Race/Ethnicity, social justice, social connectedness

## Abstract

We examined whether race/ethnic-specific social cohesion is associated with race/ethnic-specific HIV diagnosis rates using Bayesian space-time zero-inflated Poisson multivariable models, across 376 Census tracts. Social cohesion data were from the Southeastern Pennsylvania Household Health Survey, 2008—2015 and late HIV diagnosis data from eHARS system, 2009—2016. Areas where trust in neighbors reported by Black/African Americans was medium (compared to low) had lower rates of late HIV diagnosis among Black/African Americans (Relative Risk (RR)=0.52, 95% credible interval (CrI)= 0.34, 0.80). In contrast, areas where trust in neighbors reported by Black/African Americans were highest had lower late HIV diagnosis rates among Whites (RR=0.35, 95% CrI= 0.16, 0.76). Race/ethnic-specific differences in social cohesion may have implications for designing interventions aimed at modifying area-level social factors to reduce racial disparities in late HIV diagnosis.

## 1. Introduction

Neighborhood economic and social factors influence patterns in HIV incidence and diagnosis in the population (Zierler et al., 2000; Buot et al., 2014). For instance, socioeconomic factors such as poverty and unemployment reduces individual’s ability to access material resources for basic survival and creates power imbalances to negotiate safe sex, which increases susceptibility and overall HIV risk (Johnston, 2013; Brawner et al., 2017) including poor virologic outcomes (e.g. low CD4+ count) (Shacham et al., 2013). At the area level, socioeconomically deprived neighborhoods typically have fewer material and physical resources and higher density of crime markets (Latkin et al., 2013; Poundstone et al., 2004), which contribute to disparities in HIV infection rates (Friedman et al., 2009; An et al., 2013).

Aside from economic and built environment factors, the social characteristics of areas also influence patterns of HIV risk (Auerbach et al., 2011) and racial disparities in HIV diagnoses (Adimora and Schoenbach, 2002). Neighborhood social capital is defined often as collective resources and opportunities embedded within a social network that residents can access, as well as features that make it possible to achieve coordinate actions (Kawachi et al., 2004; Kawachi and Berkman, 2014). Social cohesion describes the psychological and attitudinal aspects such as perceptions of trust, feelings of belongingness and reciprocity, which are interconnected with the structural aspects of social capital (Fonseca et al., 2018; Harpham, 2008). Social capital may influence areal-level HIV diagnosis dynamics, for instance, if residents can mobilise to lobby for more HIV testing sites and other prevention resources, which may increase timely preventive health risk screenings. On the other hand, one drawback of social capital mobilization may be that some residents with more political or economic power lobby to stop or remove prevention resources they perceive as undesirable (Takahashi, 1998). Psychological appraisals of one’s neighbors such as perceived degree of belongingness, helpfulness of and trust among residents also influence area-level patterns of HIV risk and diagnosis. Sharing of information is one mechanism through which trust in neighbors can facilitate rapid spread and delivery of timely correct HIV prevention information (Cene et al., 2011; Nguyen et al., 2015). However, the proliferation of misleading or incorrect information within a community may jeopardize HIV prevention (Daskalakis et al., 2019). People working together to change stigmatizing social norms is another way that area-level social cohesion may influence HIV diagnosis dynamics (Sivaram et al., 2009; Nhamo-Murire et al., 2014).

Many studies have empirically demonstrated links with social capital and social cohesion and individual outcomes including HIV testing and disclosure of one’s HIV status (Karim et al., 2008; Grover et al., 2016). There are only a handful of ecological studies linking these factors to late HIV diagnosis rates (Y Ransome et al., 2017; Y Ransome et al., 2016; Y Ransome et al., 2016)—the outcome in this study. Population rates of late HIV diagnosis (or Stage 3 AIDS)—presenting AIDS within 90 days of an initial HIV diagnosis, is critical because earlier diagnosis improves people’s opportunities to live longer (Girardi and Sabin, 2007). On aggregate, if individuals’ viral load becomes controlled through early initiation of Antiretroviral Therapy (ART) (Tanser et al., 2013), transmission risk within the neighborhood is lowered (Wilson et al., 2008). In 2017, nationally, 45% of newly diagnosed HIV was late (Centers for Disease Control and Prevention 2017) and these disturbing trends of late diagnosis are expected to persist (Xia et al., 2015). Racial disparities in late HIV diagnosis are stark. The U.S. average rate of late HIV diagnosis in 2019 was 37.3 per 100,000 for Black/African Americans compared to 4.6 per 100,000 for non-Hispanic Whites (Centers for Disease Control and Prevention 2019).

While researchers have examined how neighborhood social capital and social cohesion are associated with late HIV diagnosis rates, those studies relied on cross-sectional data at a single time. Only one published study examined social cohesion and race/ethnic differences in late HIV diagnosis and stratified HIV diagnosis rates by race. Yet, that study did not disaggregate social cohesion by race (Y Ransome et al., 2017). Examining social cohesion disaggregated by race/ethnicity is important for us to monitor what additional social resources, beyond health care system factors, potentially contribute to lowering the burden of HIV among Black and Hispanic/Latino groups. Stratifying social capital and social cohesion by race can also allow us to identify the extent to which resources from other racial groups (Orr, 1999) influence health, which we call a “spillover effect of social capital.”

We investigate whether there is a global association of social cohesion or spillover effect of race/ethnic-specific social cohesion on race/ethnic-specific late HIV diagnosis rates, over time. Race/ethnicity is one axis of stratification that affects the quality, creation, access, look, feel, and quantity of social capital and social cohesion (Layer et al., 2014; Gilbert and Dean, 2013; Hobson-Prater and Leech, 2012; Stolle et al., 2008; Marschall and Stolle, 2004), which on aggregate will be differently distributed within an area (Marschall and Stolle, 2004).

## 2. Methods

### 2.1. Study setting

The study region is Philadelphia, PA, which is a major diverse city in the Northeast region of the U.S. In 2019, Philadelphia had an estimated population of 1580, 863 residents, about 41% of its population was Black or African American, 35% Non-Hispanic White, and 15% Hispanic or Latino. The average median income of its residents was $39,759 corresponding to 26% of the population below the Federal Poverty Level. The income distribution, however, is highly spatialized and unequally distributed ranging from $106,823 to $15,232 across neighborhoods (The PEW Charitable Trusts 2019). As of cases reported through June 2019, 18% of newly diagnosed HIV in the city were considered late. The highest HIV incidence are among men, persons aged 25–34, and men who have sex with men. HIV prevalence is highest among non-Hispanic Blacks, followed by Hispanic. One important good note is that 86.1% of people diagnosed with HIV were linked to care and 49.5% were virally suppressed (Higgins et al., 2019).

### 2.2. Outcome variable and HIV population

Late HIV diagnosis is an AIDS-defining illness or CD4+ count of <200 cells/uL within three months of an initial HIV diagnosis as reported in the Enhanced HIV/AIDS Reporting System (eHARS) to the Philadelphia Department of Public Health AIDS Activities Coordinating Office. The outcome variable was the annual number of late HIV diagnosis stratified by race/ethnicity (White, Black/African American, and Hispanic/Latino) and census tract among adults aged 18 years and older between January 1st, 2010 and December 31st, 2016. At the time of the study, only HIV data through 2016 were available. Descriptive statistics of the outcome variable is provided in Table A1 in the Appendix. In total, there were 452 late HIV diagnoses.

### 2.3. Exposure variables (Social capital and social cohesion)

Those variables were from the Southeastern Pennsylvania Household Health Survey (SPHHS), which is conducted biennially. Years, 2008, 2010, 2012, and 2015 were selected to have one-year lag preceding the outcome variable. At the time of the study analysis, 2015 was the latest year of SPHHS data available. Briefly, the SPHHS is a random-digit dialing comprehensive telephone and cellphone survey that collects population responses about health, social, economic and behavioral items among approximately 7500 adults age 18 years and older in Bucks, Chester, Delaware, and Philadelphia counties (≈30,000 adults) (Public Health Management Corporation Community 1983). Data were restricted to Philadelphia county. We explored a social cohesion composite index from three indicators (1) feelings of belongingness, derived from the question “I feel that I belong and am a part of my neighborhood?” 2) trust in neighbors, derived from the question “most people in my neighborhood can be trusted,” 3) neighbors willing to help, derived from the question “please rate how likely people in your neighborhood are willing to help their neighbors with routine activities such as picking up their trash cans, or helping to shovel snow.”) Next, we examined indicators of social capital: 4) civic and social participation, which is a count, in response to the question “how many local groups or organizations in your neighborhood do you currently participate in such as social, political, religion, school-related or athletic organization?” and 5) collective engagement, from the question “have people in your neighborhood ever worked together to improve the neighborhood?” Finally, in the multivariable analyses, we also analyzed each social capital and social cohesion indicators independently (i.e., together in one model) to identify which indicators are most salient for interventions to reduce late HIV diagnosis.

To produce tract-level measures across the 376 tracts, for each outcome for each year, we followed the approach of Quick and colleagues (Quick et al., 2020). Briefly, the statistical model is an analysis of the individual-level survey responses and estimated tract-level measures corresponding to each question by accounting for spatial-, temporal-, and between race/ethnicity and gender sources of dependence in addition to city-level variation by age and by poverty status. These demographic-specific estimates were then population weighted to produce race/ethnic-specific, tract-level estimates that are age/gender-adjusted and reflect the underlying poverty rates. The average number of survey respondents per biennial survey was *n* = 1764 White, *n* = 1685 non-Hispanic Black, and *n* = 383 Hispanic/Latino. The goal of the spatio-temporal modeling was not to produce precise estimates of these various tract-level measures for each combination of race, sex, age, and poverty level for each of the SPHHS years, but rather to produce an estimate of these measures along with measures of their uncertainty that reflects the amount of data (or lack thereof) upon which they are based, where no alternate measures currently exist (Quick et al., 2020).

### 2.4. Covariates

Control variables were selected based on theory and prior work on the topic (Buot et al., 2014; Y Ransome et al., 2016; Holtgrave and Crosby, 2003; Sampson, 2012). Continuous distributions of the following variables were included: the percentage of males, proportion of the population that are Black or African American alone; the proportion of foreign-born persons; the proportion of vacant housing in the area; and a principal component index of neighborhood socioeconomic disadvantage comprised of the percentage of poverty, the percentage of educational attainment (with a degree equivalent to or higher than high school certificate), the percentage of unemployment, and median income reverse coded.

### 2.5. Statistical analysis

We use space-time Zero-Inflated Poisson (ZIP) models to analyze the late HIV diagnosis counts by race/ethnicity, accounting for the excess zeros. Trends of race/ethnicity stratified late HIV diagnoses were estimated in the ZIP model with year as a covariate. A ZIP model is a two-component mixture model with a Bernoulli distribution modeling zeros and a Poisson distribution modeling non-zeros. In our analysis, a ZIP model is structured in Eqs. (1) and (2), where ***Y***_***itk***_, ***π***_***itk***_ and ***λ***_***itk***_ refer to the observed late HIV diagnosis count, the probability of observing a positive count, and the expected late HIV diagnosis rate in area ***i*** at year ***t*** among ethnicity ***k***., respectively. ***n***_***it***_ is the total population in the ***i^th^*** area at year ***t. λ***_***itk***_ is further linked with the covariates, including socio-demographic, socioeconomic, and social capital and cohesion variables (denoted as ***X***_***itk***_ with corresponding coefficient vectors ***β***_***k***_). Additionally, ***α***_***k***_ is the overall late HIV diagnosis rate among ethnicity ***k; ψ***_***ik***_ accounts for the geographical variations not captured by the covariates, which is spatially unstructured; ***γ***_***k***_ is the city-level trend of late HIV diagnosis in Philadelphia among ethnicity ***k***; and ***δ***_***ik***_ is the spatially unstructured differential trend at each area that deviates from the city-level trend. Details on prior specifications for the unknown parameters are provided in the Appendix.


(1) $$Pr\left(y=Y_{itk}\right)=\left\{\begin{array}{l} 1-\pi_{itk}+\pi_{itk}*Poisson\left(0|\lambda_{itk}*\;n_{it}\right)\left(Y_{itk}=0\right)\\ \;\pi_{itk}*Poisson\left(Y_{itk}|\lambda_{itk}*\;n_{it}\right)\left(Y_{itk}>0\right) \end{array}\right.$$

(2) $$\log\left(\lambda_{itk}\right)=\alpha_{k}+\beta_{k}X_{itk}+\psi_{ik}+\left(\gamma_{k}+\delta_{ik}\right)*t$$

All the ZIP models were implemented with Bayesian approaches via the algorithm Integrated Nested Laplace Approximation (INLA, available in the R package ***INLA***) (Blangiardo et al., 2013) a more computationally different alternative to the traditional Markov Chain Monte Carlo (MCMC) algorithm to implement Bayesian models. We also fitted models with other prior options for the geographical variations ***ψ***_***ik***_ (i.e., a convolutional model with both spatially structured and unstructured effects), the city-level trend ***γ***_***k***_ (i.e., a first-order autoregressive rather than a linear trend), and the differential trend ***δ***_***ik***_ (i.e., spatially structured rather than unstructured) to identify the best-fitting models. Model comparison was based on the Deviance Information Criterion (DIC) value. A lower DIC value indicates better model fit. Beta (B) coefficients are reported for the trend analysis and Relative Risks (RR) and 95% Credible Intervals (CrI) are reported for the multivariable analyses.

Lastly, we produced trend graphs using predicted estimates from regression models to show how social capital and social cohesion indicators change over time (Appendix Figure 1) and how late HIV diagnosis rates vary over time (Appendix Figure 2).

### 2.6. Ethical considerations

Institutional ethics review boards from Philadelphia Department of Public Health and Yale School of Public Health reviewed and approved the study and procedures.

## 3. Results

### 3.1. Descriptive

Social capital and social cohesion scores declined between 2008 and 2015 across all groups but the decline was significant among White and Hispanic/Latinos only (Appendix Figure 1 and coefficients displayed in Appendix Table 1). Feelings of belongingness declined significantly among Black/African Americans and Whites only. Trust among neighbors declined significantly across all groups but was steepest among Black/African Americans.

The rates of late HIV diagnosis declined significantly across all groups (Appendix Figure 2). The rate of decline was steepest for Hispanic/Latinos (regression coefficient for significance in the trends [B]= −0.35, 95% CrI: −0.47, −0.24), then for Whites *B* = −0.25, 95% CrI: −0.36, −0.15), then for Black/African Americans (*B*= −0.19, 95% CrI: −0.25, −0.13) (coefficients displayed in Appendix Table 2).

### 3.2. Association between global social capital and social cohesion with race/ethnic-specific late HIV diagnosis rates

No clear patterns emerged when social cohesion variables were examined individually. The reference group for each social capital and social cohesion indicators is “low” levels. Overall, medium levels of working to improve the community was positively associated with late HIV diagnosis rates among Black/African Americans. High levels of neighbors willing to help each other were negatively associated with late HIV diagnosis rates among Whites and Hispanic/Latinos. Medium levels of trust in neighbors among Hispanic/Latinos was negatively associated with late HIV diagnosis among Hispanic/Latinos (Table 1 contains the coefficients and the 95% Credible Intervals). A summary of the directions of association are in Appendix Table 4.

### 3.3. Spillover effects of race/ethnic-specific social capital and social cohesion associations with race/ethnic-specific late HIV diagnosis rates

There was some but inconsistent indications of spillover associations between social cohesion and race/ethnic-specific late HIV diagnosis rates (Tables 1 and 2). Areas with high (compared to low) trust among neighbors reported by Black/African Americans had lower late HIV diagnosis among Whites (RR = 0.35, 95% CrI: 0.16, 0.76).

Areas with medium (compared to low) civic and social participation reported by Whites had lower late HIV diagnosis rates for Hispanic/Latinos (RR = 0.55, 95% CrI: 0.31, 0.95). Areas with medium (RR = 0.55, 95% CrI: 0.34, 0.87) and high (RR = 0.58, 95% CrI: 0.35, 0.97) levels of neighbors helping neighbors reported by Hispanic/Latinos had lower late HIV diagnosis rates among Whites.

There were a few associations between social cohesion and late HIV diagnosis within race/ethnic groups. Areas with medium (compared to low) trust among neighbors reported by Black/African Americans had lower late HIV diagnosis for Blacks (RR = 0.52, 95% CrI: 0.34, 0.80). Areas with high (compared to low) levels of neighbors helping others reported by Whites had lower late HIV diagnosis for Whites (RR = 0.44, 95% CrI: 0.18, 0.88). No such associations were found among Hispanic/Latinos.

## 4. Discussion

Race/ethnic-specific distributions in social cohesion may have implications for designing community-level interventions aimed at modifying social factors to reduce racial disparities in late HIV diagnosis. We find that trends in social capital and social cohesion are not stable, which is in contrast to one country-level study (Dragolov et al., 2016). Instead, we found declining trends, which is consistent with trends observed in one previous other U.S. study spanning multiple geographies (Kaiser et al., 2016) and previous national trends observed by Robert Putnam in his *Bowling Alone* study (Putnam, 2000). We advance how social capital potentially influences population health through evidence that indicates a potential ‘spillover effect’ as well as showing that race/ethnic-specific associations matter.

Social capital and social cohesion resources and distributions vary by race/ethnicity because of historical racism policies such as residential segregation, redlining, and economic disinvestment (Hobson-Prater and Leech, 2012). Long-term residence in under-resourced segregated communities creates psychological shocks on residents that can paralyze their ability to achieve group-level social cohesion (Fullilove, 2013; Fullilove, 2004). We are not aware of any empirical published study that examined whether race/ethnic specific distributions of social capital and social cohesion influence race/ethnic distributions in health. There is, however, strong historical and contemporary evidence to indicate that social and economic mobilization among Black people is an important strategy to combat deficient medical care, medical abuse, race-based discrimination, and economic marginalization (Gamble, 1995; Nelson, 2011). Unfortunately, net economic and other resources; social capital and cohesion among Black people appears insufficient to improve health and social conditions among them (Orr, 1999; Gamble, 1995). This is because economic, political, and social resources including social capital provided by Whites often influence the level of social capital available to Blacks, thus producing both a direct and indirect association with the health and social plight of Black people (Gilbert et al., 2022). Moreover, Black people, when further stratified by sex, have differences in social capital access and there is often racial homogeneity in social capital. For instance, one social network study conducted in Charlotte, NC found that Black men in their sample had only one person they could go to for tangible support, such as advice or connections for jobs and housing. White people had a larger network but less diverse (i.e., mostly comprised of White people) while Black people had a moderate network but partially diverse, and Latinos had the smallest network but also partially diverse (Busette et al., 2020).

Our findings highlight the importance of investigating race/ethnic-specific social capital and social cohesion when possible (Gilbert et al., 2022). We suggest that these results be used to identify gaps within and across neighborhoods that need to be bridged to connect people in a common response to improving everyone’s health (Fullilove, 2013; Busette et al., 2020). Our results provide one starting point for assessing the degree of intergroup or bridging social capital needed to improve health for many groups. So far, lower late HIV diagnosis rates among Whites seem to be influenced by social cohesion and social capital among Whites, Blacks, and Hispanics. In contrast, late HIV diagnosis among Blacks or Hispanics was negligibly influenced by social capital or social cohesion from those outside their race. These results partially suggest that race may not always moderate the relationships between social capital and outcomes such as HIV or health which was something found in one study conducted among adults in South Africa (Olamijuwon et al., 2018).

The association we found with trust (although not observed across all levels) may shed light to emerging work indicating that trust among neighbors appears relevant for reducing late HIV diagnosis among Black/African Americans. Specifically, one ecological study in the U.S. examined late HIV diagnosis rates and social trust—operationalized as mean aggregated responses of yes to the question, “if you lost a wallet or purse that contained two hundred dollars and it was found by a neighbor, do you think it would be returned with the money in it?” The authors found the strongest negative associations between social trust and late HIV diagnosis rates among Black/African Americans (Y Ransome et al., 2017). In our study, trust refers to the question, “most people in my neighborhood can be trusted.” In a previous study conducted in Philadelphia, PA, the authors found that Black people were more likely to trust people in their neighborhood who were long-time residents—who are considered insiders, compared to people who were recent residents—considered as outsiders (BM Brawner et al., 2015). Building trust in communities of color could start with identifying people who have institutional memory of the community and are long-time residents. Future avenues for research may include developing psychometrically robust, culturally relevant and specific measurement of trust (and other social cohesion indicators) to study in association with HIV outcomes (Friedman et al., 2007; Friedman et al., 2020). The research should also identify places in the neighborhood that can cultivate or promote trust in one’s neighbor, if found to be relevant for other health outcomes, since HIV is a relatively rare outcome. Barbershops and beauty salons have been identified as important spaces where social capital is cultivated and that can be conduits for HIV prevention interventions to reduce HIV among Black people (Wilson et al., 2014). Further work is also needed to identify other spaces for building social capital and social cohesion that are salient for White and Hispanic/Latino groups.

High levels of neighbors reporting willingness to help with routine tasks and participation in local community groups by Hispanic/Latinos was associated with lower late HIV diagnosis among Whites. What is striking however, is that the decline in the crude rate of late HIV diagnosis was strongest among Hispanics but not significantly related to any of the social cohesion variables in adjusted models. We anticipated some significant associations based on prior work showing how Latinos leveraged their social capital to improve their socioeconomic outcomes in their neighborhoods (Small, 2004) and other work documenting resources that some Latino HIV+ women used to survive and thrive while living with HIV in low-income neighborhoods (Chase, 2011). It is possible that we did not find significant associations because social cohesion remained relatively stable among Hispanic/Latinos during our study period. Further ethnographic research is necessary to understand what social and economic factors account for declines in late HIV among Hispanic/Latinos.

There are some limitations to this work. Although we controlled for well-known area-level covariates, unobserved confounding remains. Crime, income inequality, and other social factors such as family fragmentation or racial diversity index could potentially minimize or mediate the impact of social cohesion on late HIV diagnosis. Many of those risk factors though are influenced by structural racism, for which ecological-level measures are now emerging (Dougherty et al., 2020). Next, there is no generally accepted way to specify social cohesion in regression models. We accounted for non-linearities between social capital and social cohesion with late HIV diagnosis using tertiles of low, medium, and high, but based the cut points on the population distribution in this sample, which will vary across other geographic boundaries (e.g., ZIP code, county). These choices could produce different results, which is related to the modifiable areal unit problem that is, the associations we found at the census tract may not exist at the ZIP code or other geographic and administrative levels. Therefore, future studies need to replicate this analysis at other levels. Nevertheless, focusing on census tracts (the smallest level of geography available for HIV data) is a strength because the areas are small enough to direct focused resources. For instance, public health officials can use clustering techniques identified in previous work to target for social cohesion interventions in areas with high HIV incidence and low social capital (Y Ransome et al., 2017; BM Brawner et al., 2015). Small area estimates of social cohesion by race/ethnicity, time, and census tract has not been previously available, although the technique itself has been used to produce prevalence estimates over time of several variables in national data such as the Behavioral Risk Factor Surveillance Survey (BRFSS) (Zhang et al., 2015). There is no baseline to identify the extent of measurement error present through this multi-year ecological analysis, which is in part, reflected in the wide confidence limits of the social cohesion by race/ethnicity trend graphs. For this analysis, we modeled the median point estimate only. Nevertheless, these social capital and social cohesion measures were constructed by methodologically rigorous Bayesian spatial techniques that have been applied to and validated with health outcomes in the SPHHS (Quick et al., 2020). While we used widely accepted measures, there may be culture-specific indicators (e.g., block parties; lending groups, informal negotiations) that are more salient for each racial/ethnic group (Friedman et al., 2007; Dean et al., 2015; Sanyal, 2009). It was not possible to further examine these data stratified by sex because those data were not available. In Philadelphia, PA, rates of HIV are highest among men and findings suggest that the association between social capital and HIV-related risk factors vary by sex even within race and ethnicity (Ransome et al., 2020), and so may possibly exist for HIV-outcomes as well. Last, there is potential for statistical biases due to multiple race/ethnic-specific models predicting each race/ethnic-specific HIV outcome. However, the Bayesian statistical framework and procedures that we used, which incorporates Priors, overcomes type S error/multiple comparisons problem that is present in classical frequentist regression (Gelman and Tuerlinckx, 2000; Gelman et al., 2012). Last, these findings are based on ecological-level analysis, and so inference is limited only to aggregate-level representations of social capital and social cohesion, thus we cannot assume or infer that these relationships operate among individuals in similar fashion.

Despite these potential limitations, to our knowledge, this is the first study to quantify and show that race/ethnic-specific social cohesion has differential impacts on race/ethnic-specific late HIV diagnosis rates. Some strengths of our study include nine years of cross-sectional serial ecological data for social capital and social cohesion and HIV diagnosis rates. We need to better integrate the ‘social’ in Social Determinants of Health (Holt-Lunstad, 2022) to address health inequalities, and this study provided some evidence that can move these conversations forward. Some examples of current efforts, as of year 2020, that leverage community social cohesion to improve population health through reducing racial and socioeconomic inequalities (Fullilove, 2020; Ransome et al., 2021) are “Invisible Hands” and Bed-Stuy Strong where volunteers deliver groceries and other essential items to vulnerable members within neighborhoods. If our findings hold in replicate analyses across other cities, public health agencies may consider interventions to partner with these groups to deliver health programming through community building activities (Ransome and Ritchwood, 2020).

## Supplementary Material

Supplement

## Figures and Tables

**Table 1:** Associations Between Aggregate Social Capital and Social Cohesion and Race/Ethnic-Specific Late HIV Diagnosis.

Social capital and social cohesion by all groups on aggregate	Late HIV diagnosis among Black/African Americans			Late HIV diagnosis among Whites			Late HIV diagnosis among Hispanic/Latinos		
	**Relative Risk and 95% Credible Interval**								
	RR	95% CrI		RR	95% CrI		RR	95% CrI	
% of male	0.96	0.83	1.11	0.85	0.66	1.08	1.00	0.85	1.16
% of black people	2.49	2.00	3.11	0.54	0.41	0.70	0.79	0.62	1.01
% of foreign born	1.18	0.97	1.43	0.97	0.80	1.17	1.02	0.83	1.24
% of vacant homes	1.08	0.92	1.25	0.96	0.79	1.17	0.99	0.79	1.23
Socioeconomic disadvantage	1.29	1.03	1.61	1.04	0.80	1.35	1.86	1.46	2.38
Feelings of belongingness (med)	0.88	0.59	1.32	0.89	0.54	1.46	1.02	0.66	1.57
Feelings of belongingness (high)	1.08	0.63	1.85	1.00	0.51	1.98	0.84	0.38	1.86
Trust in neighbors (med)	0.86	0.60	1.23	1.23	0.68	2.19	0.47	0.25	0.85
Trust in neighbors (high)	0.70	0.41	1.21	1.42	0.61	3.27	1.26	0.51	3.04
Collective engagement (med)	1.59	1.02	2.51	1.50	0.95	2.39	0.81	0.51	1.27
Collective engagement (high)	1.40	0.84	2.34	1.71	0.96	3.05	1.06	0.58	1.94
Neighbors willing to help (med)	1.30	0.89	1.88	0.67	0.40	1.13	0.91	0.54	1.52
Neighbors willing to help (high)	1.36	0.81	2.28	0.45	0.23	0.88	0.40	0.17	0.94
Civic and social participation (med)	0.92	0.65	1.29	0.76	0.44	1.30	1.07	0.63	1.80
Civic and social participation (high)	1.06	0.70	1.62	0.84	0.45	1.58	1.02	0.51	2.00

**Notes:** The reference group for all the social cohesion and capital variables is “low” so results are interpreted as medium or high levels (compared to low levels). The models are based on multivariable analyses and all the covariates are continuously specified so there is no reference group but interpreted as a one-unit change on the standard deviation scale. “By all groups on aggregate” refers to the global indicator where race/ethnicity is not disaggregated.

*Bold font are statistically significant associations.

**Table 2:** Associations Between Race/Ethnic-Specific Social Capital and Social Cohesion and Race/Ethnic-Specific Late HIV Diagnosis, 2009 to 2016.

	Late HIV diagnosis among Black/African Americans			Late HIV diagnosis among Whites			Late HIV diagnosis among Hispanic/Latinos		
	**Relative Risk and 95% credible interval**								
**Black social capital and social cohesion in association with late HIV diagnosis**	RR	95% CrI		RR	95% CrI		RR	95% CrI	
% of male	0.97	0.84	1.13	0.87	0.67	1.11	1.03	0.85	1.25
% of black	**2.45**	**1.96**	**3.08**	**0.59**	**0.45**	**0.78**	**0.68**	**0.51**	**0.90**
% of foreign born	1.14	0.94	1.37	0.94	0.77	1.14	1.07	0.85	1.35
% of vacant homes	1.06	0.91	1.24	1.01	0.83	1.23	0.99	0.77	1.26
Socioeconomic disadvantage	1.20	0.97	1.49	0.88	0.67	1.15	**2.04**	**1.54**	**2.71**
Feelings of belongingness (med)	1.06	0.72	1.56	0.86	0.53	1.41	0.97	0.54	1.72
Feelings of belongingness (high)	1.21	0.73	2.01	0.83	0.41	1.67	0.86	0.39	1.86
Trust in neighbors (med)	**0.52**	**0.34**	**0.80**	0.78	0.45	1.35	1.20	0.66	2.18
Trust in neighbors (high)	0.66	0.38	1.10	**0.35**	**0.16**	**0.76**	0.76	0.32	1.77
Collective engagement (med)	0.86	0.54	1.35	0.99	0.61	1.61	1.06	0.60	1.87
Collective engagement (high)	1.19	0.72	1.96	0.99	0.54	1.81	1.27	0.63	2.55
Neighbors willing to help (med)	1.00	0.73	1.37	0.84	0.52	1.36	1.13	0.66	1.94
Neighbors willing to help (high)	1.00	0.65	1.53	0.70	0.33	1.47	1.15	0.54	2.43
Civic and social participation (med)	0.91	0.68	1.23	0.96	0.61	1.52	0.80	0.49	1.31
Civic and social participation (high)	0.87	0.63	1.20	1.01	0.60	1.69	1.05	0.61	1.82
**White social capital and social cohesion in association with late HIV diagnosis**									
% of male	0.96	0.83	1.11	0.86	0.66	1.09	1.01	0.83	1.22
% of black	**2.60**	**2.08**	**3.26**	**0.57**	**0.44**	**0.74**	0.81	0.61	1.06
% of foreign born	1.18	0.96	1.45	0.92	0.75	1.12	1.06	0.83	1.36
% of vacant homes	1.08	0.92	1.26	0.93	0.75	1.14	1.01	0.79	1.29
Socioeconomic disadvantage	**1.30**	**1.04**	**1.62**	0.99	0.76	1.28	**1.77**	**1.33**	**2.36**
Feelings of belongingness (med)	0.99	0.65	1.49	0.70	0.38	1.28	0.72	0.40	1.27
Feelings of belongingness (high)	1.05	0.59	1.85	1.01	0.47	2.18	0.69	0.25	1.85
Trust in neighbors (med)	0.87	0.56	1.36	1.27	0.66	2.44	0.53	0.26	1.05
Trust in neighbors (high)	0.91	0.49	1.68	1.17	0.46	2.99	0.81	0.27	2.39
Collective engagement (med)	1.49	0.95	2.37	1.06	0.65	1.72	1.13	0.64	1.97
Collective engagement (high)	1.52	0.91	2.59	1.12	0.62	2.03	1.17	0.56	2.46
Neighbors willing to help (med)	0.97	0.59	1.58	0.74	0.39	1.43	1.05	0.52	2.08
Neighbors willing to help (high)	1.09	0.58	2.08	**0.44**	**0.18**	**0.88**	0.73	0.26	2.06
Civic and social participation (med)	1.08	0.78	1.49	0.76	0.46	1.24	**0.55**	**0.31**	**0.95**
Civic and social participation (high)	0.97	0.64	1.47	1.02	0.58	1.82	1.06	0.53	2.06
**Hispanic social capital and social cohesion in association with late HIV diagnosis**									
% of male	0.96	0.83	1.11	0.86	0.66	1.10	1.06	0.87	1.29
% of black	**2.72**	**2.18**	**3.44**	**0.60**	**0.46**	**0.79**	0.80	0.60	1.06
% of foreign born	1.12	0.91	1.37	0.99	0.81	1.21	1.02	0.80	1.30
% of vacant homes	1.08	0.93	1.26	0.95	0.78	1.16	0.99	0.78	1.25
Socioeconomic disadvantage	1.17	0.94	1.46	0.94	0.73	1.21	**1.68**	**1.27**	**2.21**
Feelings of belongingness (med)	0.97	0.73	1.32	1.02	0.64	1.61	0.94	0.59	1.51
Feelings of belongingness (high)	0.86	0.62	1.22	1.15	0.72	1.84	0.90	0.53	1.53
Trust in neighbors (med)	0.90	0.66	1.23	0.99	0.57	1.73	0.58	0.33	1.02
Trust in neighbors (high)	0.70	0.46	1.07	1.16	0.61	2.21	0.63	0.30	1.31
Collective engagement (med)	1.08	0.71	1.63	1.41	0.89	2.23	0.90	0.54	1.50
Collective engagement (high)	1.04	0.65	1.65	1.63	0.93	2.87	0.75	0.38	1.47
Neighbors willing to help (med)	1.30	0.97	1.75	**0.55**	**0.34**	**0.87**	0.78	0.49	1.23
Neighbors willing to help (high)	1.02	0.69	1.50	**0.58**	**0.35**	**0.97**	0.54	0.27	1.02
Civic and social participation (med)	0.86	0.64	1.15	0.42	0.24	0.69	1.18	0.71	1.94
Civic and social participation (high)	1.05	0.75	1.47	0.53	0.31	0.92	1.06	0.57	1.94

**Notes:** The reference group for all the social cohesion and capital variables is “low” so results are interpreted as medium or high levels (compared to low levels). The models are based on multivariable analyses and all the covariates are continuously specified so there is no reference group but interpreted as a one-unit change on the standard deviation scale.

In all analyses, there are covariates that correspond to the distribution of these social determinants among the total Philadelphia population, within each Census Tract: % black,% male,% of foreign born,% vacant homes, and socioeconomic disadvantage.
