# Supplementary material for: Is race-specific neighborhood social cohesion key to reducing racial disparities in late HIV diagnosis: A multiyear ecological study
Source: Spat Spatiotemporal Epidemiol. Author manuscript; Available in PMC 2023 Feb 10. (PMC9912753; doi:10.1016/j.sste.2022.100508)
Supplement: Supplement [file NIHMS1860618-supplement-Supplement.zip › 1-s2.0-S1877584522000314-mmc2.docx]

**Appendix Table 1**. Coefficients and 95% Credible Intervals for Trends in Neighborhood Social Cohesion index and Social Capital variables by Race/Ethnicity, 2008-2015

|  | **Coefficient** | **2.50%** | **97.50%** |
| --- | --- | --- | --- |
| Social cohesion index among Black/African Americans | -0.0009 | -0.00194 | 0.000139 |
| Social cohesion index among Whites | **-0.00073** | -0.00113 | -0.00034 |
| Social cohesion index among Hispanic/Latinos | **-0.00032** | -0.00061 | -1.98E-05 |
| Feelings of belongingness among Black/African Americans | **-0.00079** | -0.00159 | -1.43E-06 |
| Feelings of belongingness among Whites | **-0.00075** | -0.00106 | -0.00044 |
| Feelings of belongingness among Hispanic/Latinos | -0.00036 | -0.00083 | 0.000108 |
| Trust in neighbors among Black/African American | **-0.00129** | -0.00189 | -0.0007 |
| Trust in neighbors among Whites | **-0.00106** | -0.00177 | -0.00035 |
| Trust in neighbors among Hispanic/Latinos | **-0.00053** | -0.00084 | -0.00022 |
| Collective engagement among Black/African Americans | -0.00016 | -0.00276 | 0.002442 |
| Collective engagement among Whites | -0.00045 | -0.00339 | 0.002492 |
| Collective engagement among Hispanic/Latinos | 0.000679 | -0.00073 | 0.002093 |
| Neighbors willing to help among Black/African American | -0.00064 | -0.00218 | 0.000894 |
| Neighbors willing to help among White | -0.00035 | -0.00146 | 0.000751 |
| Neighbors willing to help among Hispanic/Latinos | 6.10E-05 | -9.15E-05 | 0.000214 |
| Civic and Social participation among Black/African Americans | 0.000242 | -0.0135 | 0.013986 |
| Civic and Social participation among Whites | 0.007457 | 9.18E-06 | 0.014905 |
| Civic and Social participation among Hispanic/Latinos | 0.012054 | 0.003093 | 0.021015 |

**Appendix Table 2** Coefficients and 95% Credible Intervals Trends in Race/Ethnic-specific Late HIV Diagnosis Rates, 2010-2016

|  | **Coefficient** | **2.50%** | **97.50%** |
| --- | --- | --- | --- |
| Late HIV diagnosis among Black/African Americans | -0.19 | 0.25 | -0.12 |
| Late HIV diagnosis among Whites | **-0.25** | -.036 | -0.15 |
| Late HIV diagnosis among Hispanic/Latinos | **-0.35** | -0.47 | -0.24 |

**Appendix Table 3:** Descriptive statistics of late HIV diagnosis in Philadelphia stratified by race/ethnicity and census tract, 2010-2016

|  | **Black/African Americans** | | | | **Whites** | | | | **Hispanic/Latinos** | | | |
| --- | --- | --- | --- | --- | --- | --- | --- | --- | --- | --- | --- | --- |
|  | **Mean** | **Min** | **Max** | **% zeros** | **Mean** | **Min** | **Max** | **% zeros** | **Mean** | **Min** | **Max** | **% zeros** |
| 2010 | 0.31 | 0 | 4 | 79.6 | 0.07 | 0 | 2 | 93.4 | 0.12 | 0 | 3 | 89.8 |
| 2011 | 0.24 | 0 | 3 | 82.9 | 0.09 | 0 | 2 | 92.0 | 0.09 | 0 | 3 | 93.4 |
| 2012 | 0.24 | 0 | 5 | 83.2 | 0.07 | 0 | 2 | 93.4 | 0.08 | 0 | 1 | 91.9 |
| 2013 | 0.20 | 0 | 3 | 84.1 | 0.07 | 0 | 2 | 94.0 | 0.03 | 0 | 1 | 96.4 |
| 2014 | 0.17 | 0 | 3 | 86.2 | 0.04 | 0 | 1 | 95.5 | 0.02 | 0 | 3 | 98.2 |
| 2015 | 0.14 | 0 | 4 | 88.6 | 0.03 | 0 | 2 | 97.3 | 0.03 | 0 | 1 | 97.0 |
| 2016 | 0.13 | 0 | 2 | 89.2 | 0.04 | 0 | 1 | 96.1 | 0.03 | 0 | 2 | 97.0 |

Prior specifications of the space-time ZIP model

We assigned a non-informative Normal prior with mean and precision equal to zero to ***αk*** (the default prior to intercept terms in INLA models). A prior Normal(0,) was specified to ***ψik***. The priors for ***γk*** and ***δik*** were Normal(0,1000) and Normal(0,), respectively. A uniform improper prior was specified to the standard deviation terms, and . To test if modeling results were sensitive to hyperprior specifications, we also fitted models with a positive half Normal prior Normal+∞(0, 100) to and (Gelman, 2006).

Gelman, A. (2006). Prior distributions for variance parameters in hierarchical models. Bayesian Analysis, 1(3), 515-533.

**Appendix Table 4:** Summary of Global- and Race/Ethnic-Specific Social Cohesion and Social Capital associations with Race/Ethnic-Specific Late HIV Diagnosis, 2009 to 2016

| **Social Cohesion or Capital** | **Associated with higher late HIV diagnosis rates** | **Associated with lower late HIV diagnosis rates** |
| --- | --- | --- |
| Social Cohesion Index | NR | NR |
| Trust among neighbors | NR | Reported by Non-Hispanic Black American: for Black and White |
| Neighbors willing to help | NR | By all groups on aggregate: for Whites and Hispanic/Latinos  Reported by Whites: for Whites |
| Feelings of belongingness | NR | NR |
| Collective Engagement | By all groups on aggregate: For Blacks only | NR |
| Civic and social participation | NR | Reported by Whites: for Hispanic/Latinos |
| **Notes:** The group listed before the colon represents the group reporting the social capital, and those after corresponds to the group where associations with late HIV diagnosis were observed. For instance, “Trust among neighbors reported by Black/African Americans was associated with lower late HIV diagnosis rates for Black and for Whites.”  All results are based on multivariable analyses adjusted for all covariates. “By all groups on aggregate” refers to the global indicator where race/ethnicity is not stratified. NR: No statistically significant relationship exists. | | |
