# Supplementary material for: Is race-specific neighborhood social cohesion key to reducing racial disparities in late HIV diagnosis: A multiyear ecological study
Source: Spat Spatiotemporal Epidemiol. Author manuscript; Available in PMC 2023 Feb 10. (PMC9912753; doi:10.1016/j.sste.2022.100508)
Supplement: Supplement [file NIHMS1860618-supplement-Supplement.zip › 1-s2.0-S1877584522000314-mmc1.docx]

**Appendix Figure 1:** Trends in Neighborhood Social Cohesion index and Social Capital variables by Race/Ethnicity, 2008-2015


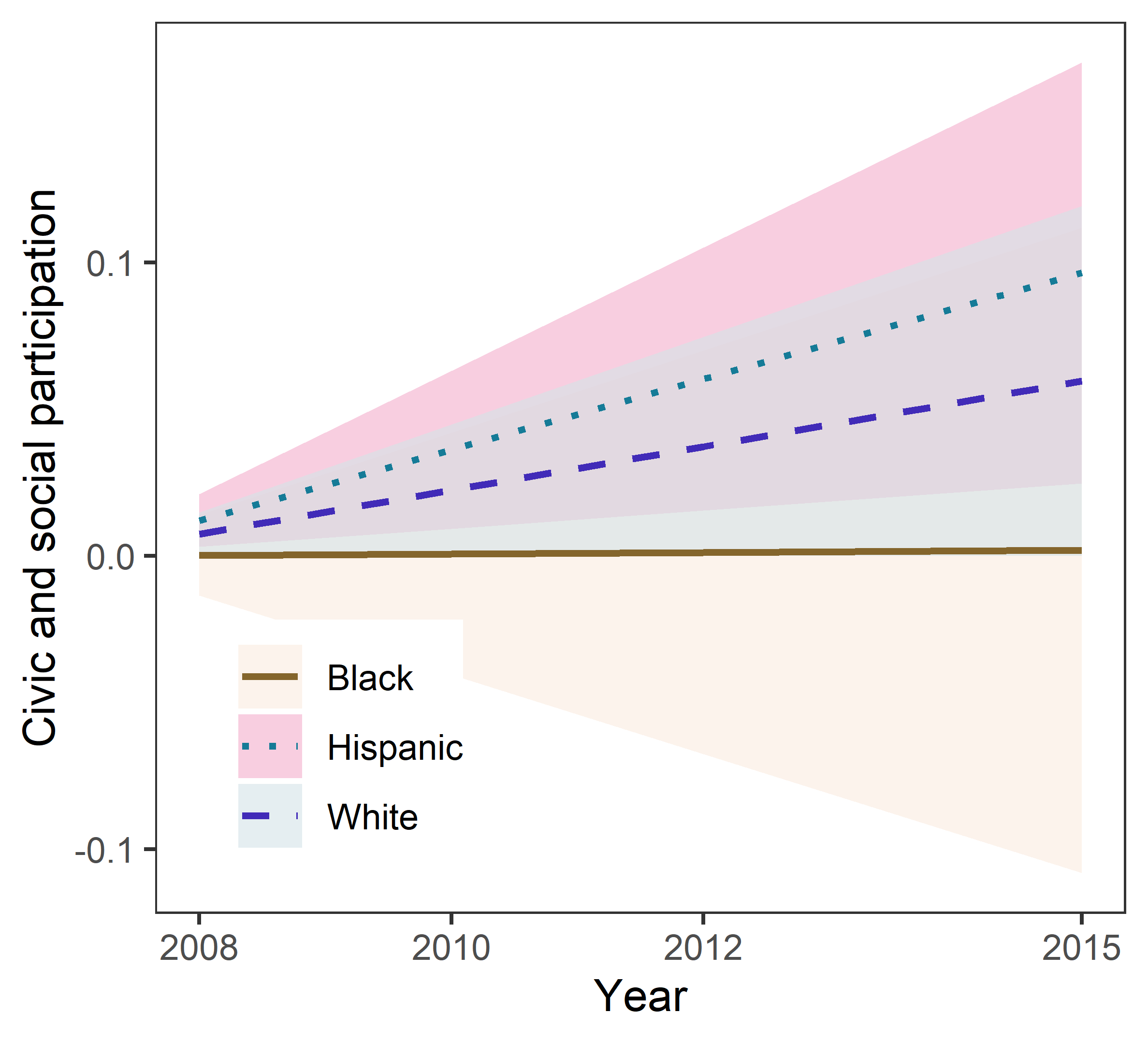

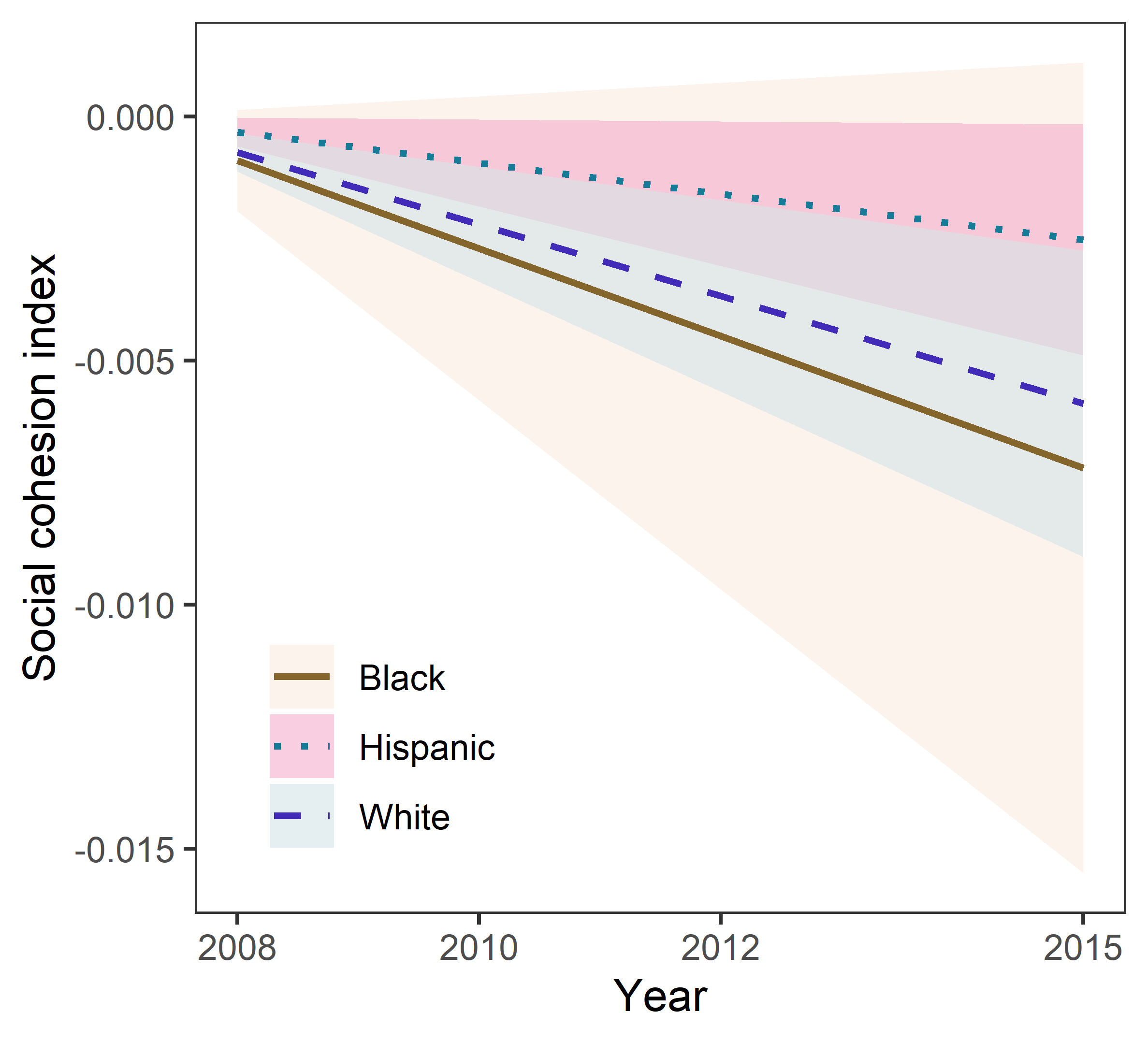

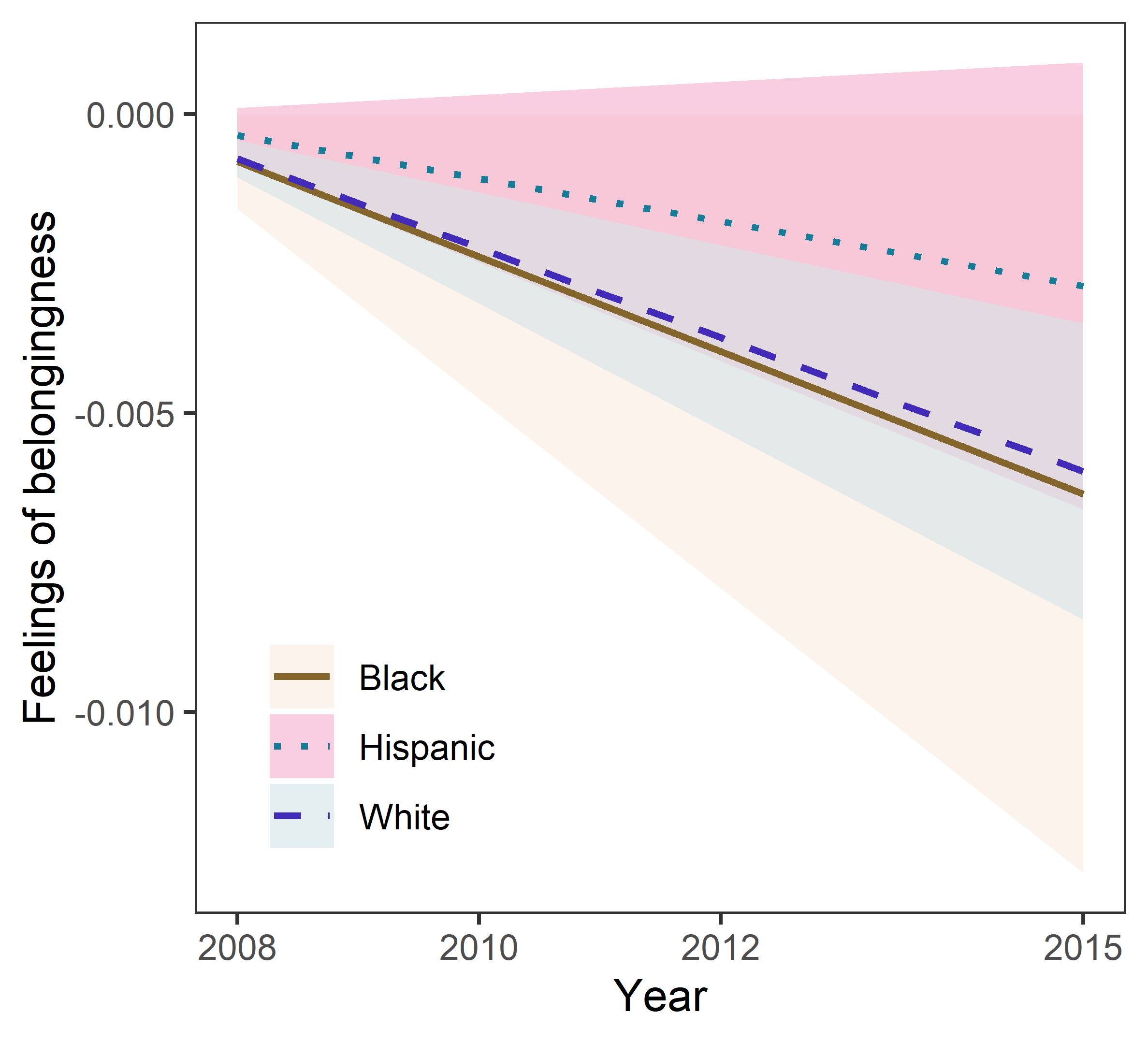

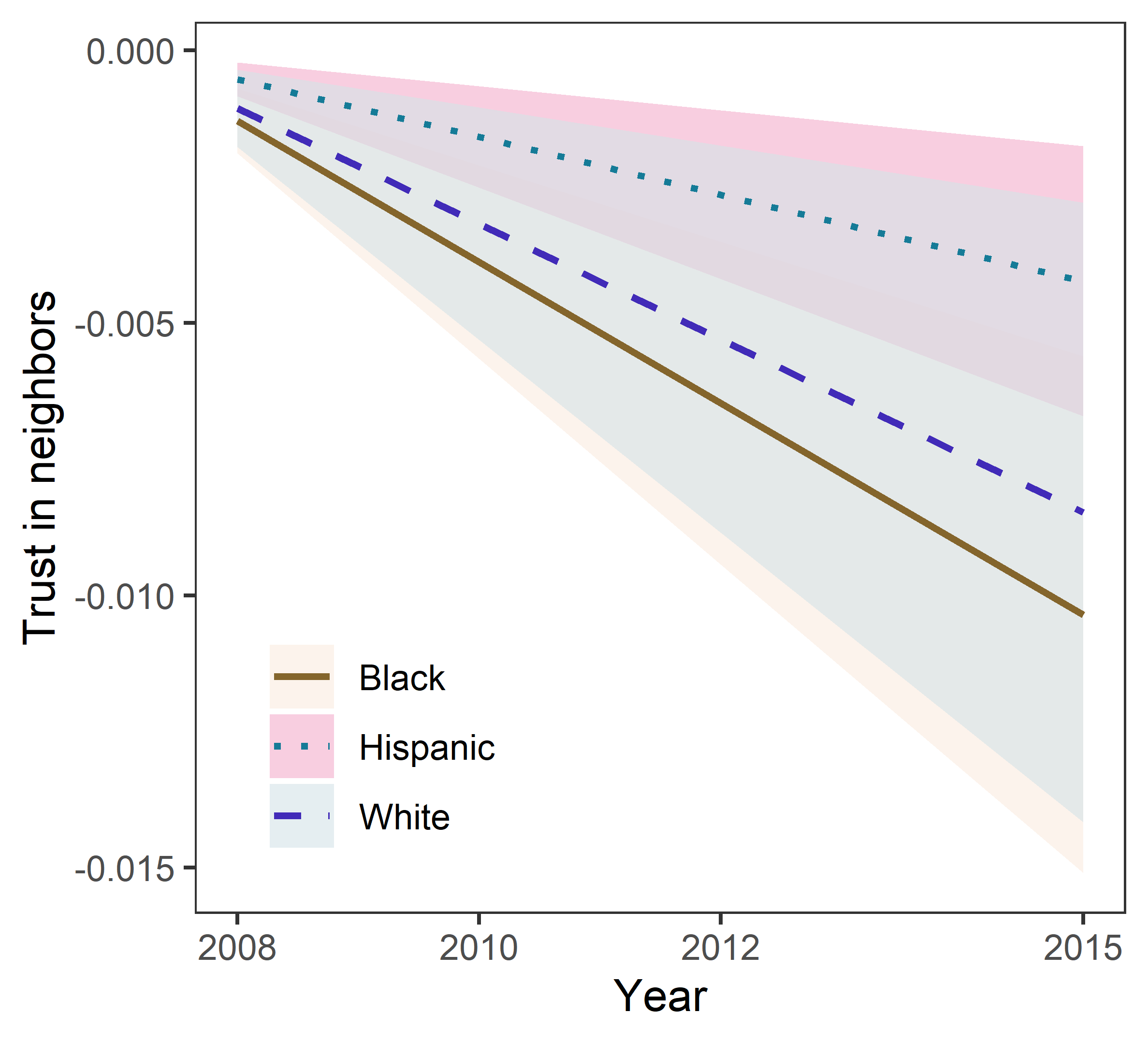

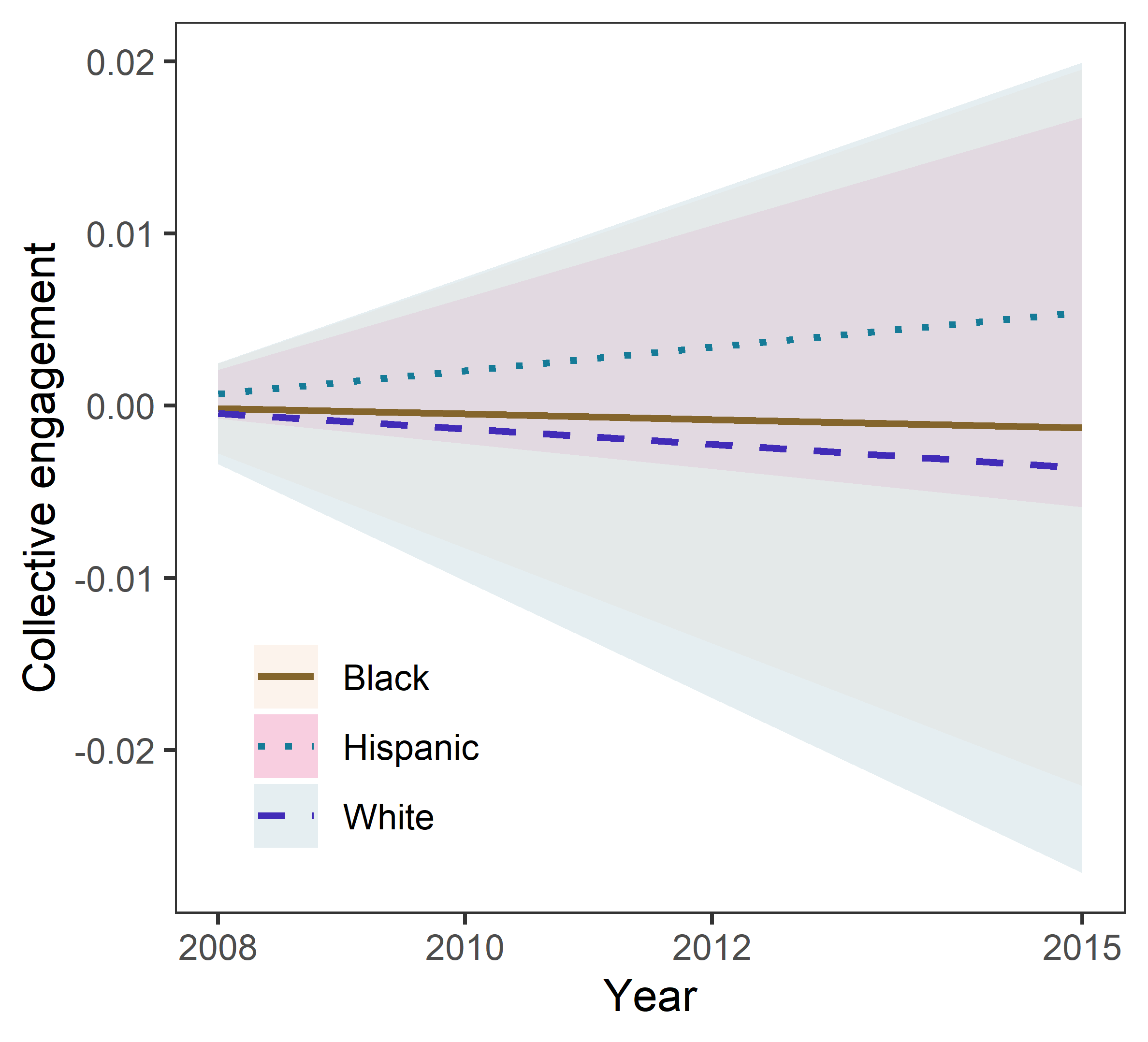

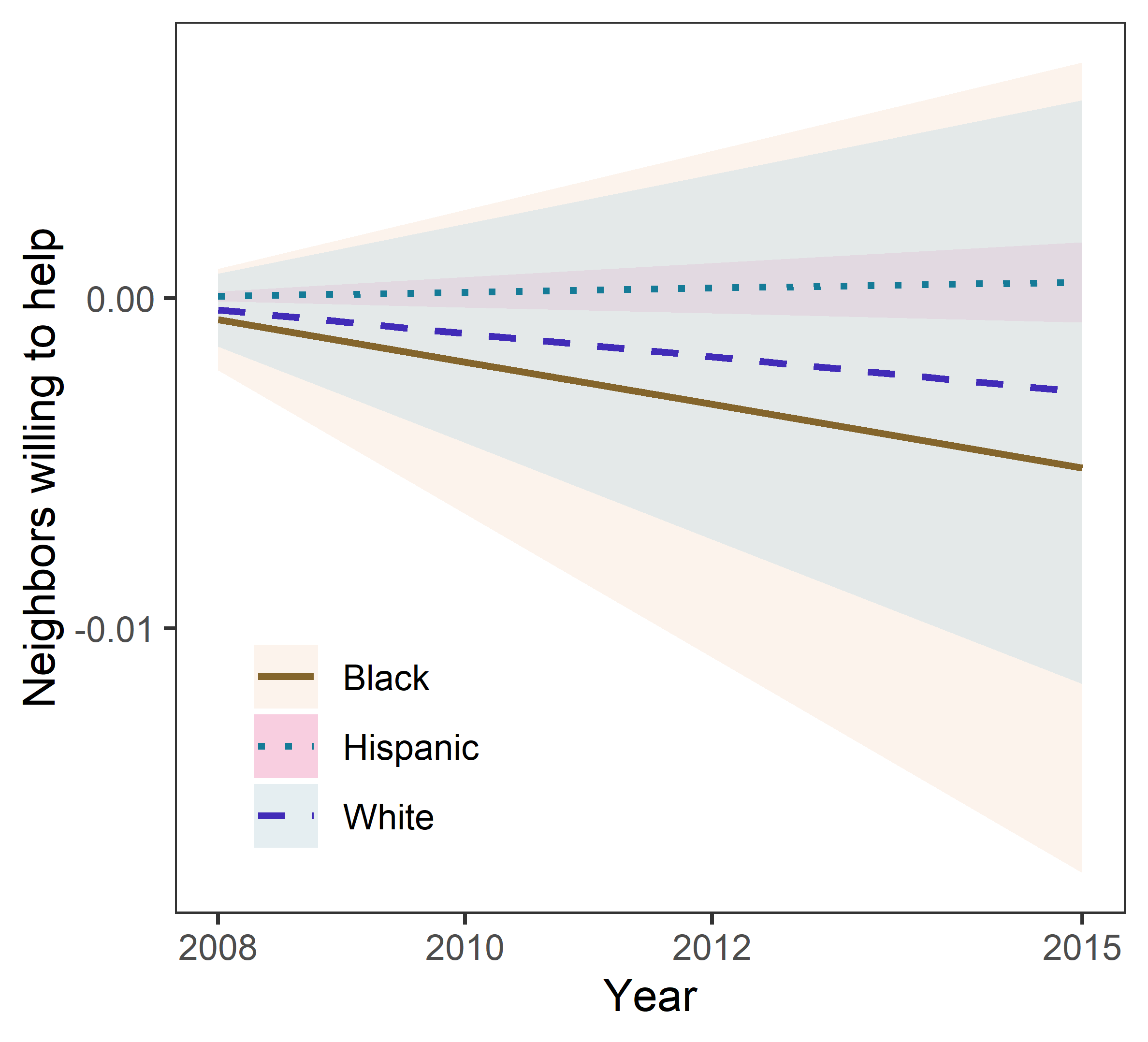


**Notes:** Lines correspond to trends and the shaded regions are the 95% Credible Intervals. Scores on the x-axis correspond to regression coefficients.


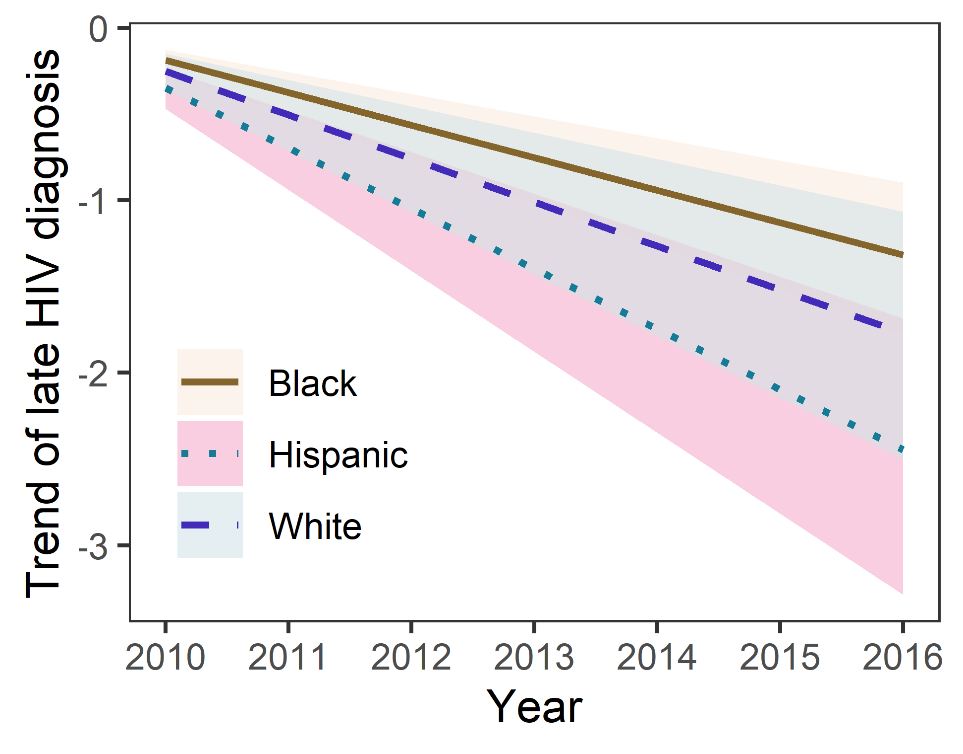
**Appendix Figure 2:** Trends in Race/Ethnic-specific Late HIV Diagnosis Rates, 2010-2016

**Notes:** Lines correspond to trends and the shaded regions are the 95% Credible Intervals. Scores on the x-axis correspond to regression coefficients.
